# Supplementary material for: Adolescent fertility and its determinants in Kenya: Evidence from Kenya demographic and health survey 2014
Source: PLoS One. 2022 Jan 12;17(1):e0262016. doi: 10.1371/journal.pone.0262016 (PMC8754288; doi:10.1371/journal.pone.0262016)
Supplement: Attachment — Submitted filename: RESPONSE TO REVIEWERS.docx [file pone.0262016.s001.docx]

To the editor,

Richard Ibañez Dilla
PLOS ONE

27^th^ October 2021

**Rebuttal Letter**

We wish to thank the academic editor and the two reviewers for their useful comments on our manuscript. All the comments and suggestions raised have been addressed. In this letter our response is indicated in bold and purple in colour. We have also included a revised manuscript with Track Changes and unmarked version without Tracked Changes. For clarification on how to access the data, the URL is provided for, under the ethics statement which is, https://dhsprogram.com/data including the data set title, KEIR72SV.ZIP. We are in agreement to the fact that interested researchers can replicate our study findings in its entirety by directly obtaining the data from the third party and following the protocol in our Methods section. In addition, we confirm that the authors did not have any special access privileges that others would not have. We also approve update of Data Availability statement on our behalf to reflect the information we have provided. Further, below is the response of all points and suggestions raised by the editor and the reviewers. We hope that we have satisfactory tackled all concerns raised and that the manuscript is now well suited for publication.

Sincerely,

On Behalf of all authors,

Naomi Monari

**Academic Editor**


Journal Requirements:

When submitting your revision, we need you to address these additional requirements.

1. Please ensure that your manuscript meets PLOS ONE's style requirements, including those for file naming. The PLOS ONE style templates can be found at 
<https://journals.plos.org/plosone/s/file?id=wjVg/PLOSOne_formatting_sample_main_body.pdf> and 
<https://journals.plos.org/plosone/s/file?id=ba62/PLOSOne_formatting_sample_title_authors_affiliations.pdf>

**We have downloaded the templates and revised the manuscript according to PLOS ONES’s style requirement. This includes formatting on title authors and the main body of the manuscript, including ensuring the manuscript is double spaced.**

2. Please provide additional details regarding participant consent. In the ethics statement in the Methods and online submission information, please ensure that you have specified (1) whether consent was informed and (2) what type you obtained (for instance, written or verbal, and if verbal, how it was documented and witnessed). If your study included minors, state whether you obtained consent from parents or guardians. If the need for consent was waived by the ethics committee, please include this information.

If you are reporting a retrospective study of medical records or archived samples, please ensure that you have discussed whether all data were fully anonymized before you accessed them and/or whether the IRB or ethics committee waived the requirement for informed consent. If patients provided informed written consent to have data from their medical records used in research, please include this information.

**We have included the Ethics statement in our manuscript which we stated that, Specific ethical approval was not required for the 2014 KDHS secondary data analysis. Consequently, a secondary analysis was done under the original consent provided by participants during the data collection process. However, permission to use the data was obtained from ICF Macro from https://dhsprogram.com/data. Their user instructions were followed, which included, treating the data as confidential and no effort should be made to identify any household or individual respondent interviewed in the survey.**

Once you have amended this/these statement(s) in the Methods section of the manuscript, please add the same text to the “Ethics Statement” field of the submission form (via “Edit Submission”).

**The ethics statement has also been included in the submission form via Edit Submission.**

For additional information about PLOS ONE ethical requirements for human subjects research, please refer to <http://journals.plos.org/plosone/s/submission-guidelines#loc-human-subjects-research>.

3. PLOS requires an ORCID iD for the corresponding author in Editorial Manager on papers submitted after December 6th, 2016. Please ensure that you have an ORCID iD and that it is validated in Editorial Manager. To do this, go to ‘Update my Information’ (in the upper left-hand corner of the main menu), and click on the Fetch/Validate link next to the ORCID field. This will take you to the ORCID site and allow you to create a new iD or authenticate a pre-existing iD in Editorial Manager. Please see the following video for instructions on linking an ORCID iD to your Editorial Manager account: <https://www.youtube.com/watch?v=_xcclfuvtxQ>

**We have followed the steps and have authenticated the pre-existing iD in the Editorial Manager**

**Additional Editor Comments:**

Dear Authors,
I would ask you to revise the paper by following the reviewers' comments and suggestions.


[Note: HTML markup is below. Please do not edit.]

Reviewers' comments:

Reviewer's Responses to Questions

**Comments to the Author**

1. Is the manuscript technically sound, and do the data support the conclusions?

The manuscript must describe a technically sound piece of scientific research with data that supports the conclusions. Experiments must have been conducted rigorously, with appropriate controls, replication, and sample sizes. The conclusions must be drawn appropriately based on the data presented.

Reviewer #1: Partly

Reviewer #2: Yes

2. Has the statistical analysis been performed appropriately and rigorously?

Reviewer #1: Yes

Reviewer #2: Yes

3. Have the authors made all data underlying the findings in their manuscript fully available?

The [PLOS Data policy](http://www.plosone.org/static/policies.action#sharing) requires authors to make all data underlying the findings described in their manuscript fully available without restriction, with rare exception (please refer to the Data Availability Statement in the manuscript PDF file). The data should be provided as part of the manuscript or its supporting information, or deposited to a public repository. For example, in addition to summary statistics, the data points behind means, medians and variance measures should be available. If there are restrictions on publicly sharing data—e.g. participant privacy or use of data from a third party—those must be specified.

Reviewer #1: Yes

Reviewer #2: Yes

4. Is the manuscript presented in an intelligible fashion and written in standard English?

PLOS ONE does not copyedit accepted manuscripts, so the language in submitted articles must be clear, correct, and unambiguous. Any typographical or grammatical errors should be corrected at revision, so please note any specific errors here.

Reviewer #1: Yes

Reviewer #2: Yes

5. Review Comments to the Author

Please use the space provided to explain your answers to the questions above. You may also include additional comments for the author, including concerns about dual publication, research ethics, or publication ethics. (Please upload your review as an attachment if it exceeds 20,000 characters)

Reviewer #1: The text is understandable, but before publishing it anywhere, it should be edited for English.

**Thank you for the comment. The manuscript has been edited for English appropriately as evidenced in the tracked changes document.**

This is an elementary analysis with very little new information. I think the importance of marriage is probably misleading, since I suspect that a great number of these were premarital pregnancies. Also the arguments for the C Index values are not convincing.

**First to the address the concern on importance of marriage as misleading this was our response:**

**The variations may have probably occurred as most of the adolescent in the current study had never been married (89.5%). It is also worth noting that the KDHS data is cross-sectional in nature and can only ascertain associations and not causality for the predictor variables under study. Hence according to Table 1 female adolescents who were married at the time of the survey constituted the majority child bearers (70.7%). While on the other hand childbearing was lowest among female adolescents who were not married (8.1%) which implied that current marital status was significantly associated with adolescent fertility.**

**Second, to address the concern for the Cm index on marriage as not convincing, the following was our response:**

**According to Bongaarts Proximate Determinants of Fertility model, female adolescent marriage index is calculated with the formula: Cm (15-19) = m (20-24)*0.75. In admitting the fact that premarital conception among teenagers are quite significant, a constant, 0.75, was introduced which Bongaarts states that the constant is used because in the age 15-19 years, the incidence of premarital conception is not negligible. Consequently, the great number of premarital pregnancies was acknowledged by Bongaarts, who introduced a constant of 0.75 to neutralize the effect and allow demographers to utilize the model to estimate fertility among the proportion of 15-19 female adolescent who were married for all populations, including urban and rural populations.**

Reviewer #2: Thank you for giving me the opportunity to review this interesting article. As it has been noted, there is a dearth of recent literature exists addressing determinates of adolescents’ fertility in the context of Kenya. I hope my comments would be helpful to increase the quality of your work.

Abstract

• Please be specific on age 15-17 years old, it could be clarified as “current age 15-17 years “

**Thank you. We have modified the manuscript in the abstract and included the words “current age” for clarity**

• Add few more Key words which are relevant to your present study.

**This has been done. We have added more key words in the manuscript which include Children Ever Born, Ordinal Regression Model and KDHS.**

Background- Include in background
• Do they have any recent studies on the determinates of fertility in the context of Kenya?

**Thank you for the observation. There exists recent literature on studies undertaken on determinants of adolescent fertility in Kenyan context which we have included to enrich the background accordingly.**

• It is not clear what do you really mean “These factors were varied from country to country and even within the 22 (line numbering) countries disparities still exist”, what sort of inequalities? Need a justification.

**Thank you. We have included a paragraph to justify the above statement, just before the statement. However, number 22 is the line numbering and does not indicate the number of countries.**

• There should be a proper discussion on what are the current research gaps in terms of fertility determinants in Kenya, it seems like jumping to the objectives without proving enough evidence/research gaps, what is the rationale and how this study could be benefited from bridge the gaps, and what would be the contribution of present study- one brief paragraph looking at this should be included.

**Thank you for the observation we have included a paragraph to explain the research gap.**

• This sentese is not clear “Exploring the factors associated with teenage childbearing ensures targeted programs to this vulnerable population sub-group for policy makers and to aid in reducing the teen pregnancy incidence” So have you used the terms “teenage and adolescents” interchangeably?

**Yes. The words teenage and adolescents have been used interchangeably in the study.**

Methods

• Methods section is clear; however, you may add an abbreviation terms somewhere for the convenience of the reader.

**Thank you. We have included a section on abbreviation just before the Acknowledgement. The abbreviations include KDHS: Kenya Demographic and Health Survey; TFR: Total Fertility Rate; TF: Total Fecundity; ASFR: Age Specific Fertility Rate; AF: Age Fecundity; OR: Odds Ratio; CI: Confidence Interval; RC: Reference Category**

• Dependent variables need to be clearer, particularly, how you have been developed some of the covariates such as wealth index, etc, do they compatible with DHS? Put a section describing variables

**Thank you. A paragraph has been inserted in the manuscript under the sub-heading “Study variable” to describe variable in the study**

Results:
• For all Tables include the sample size (n=)

**Thank you. The sample size n has been included in each Table**

• As according to the Table 3, as it has presented the disaggregation of fertility indexes by urban rural, what is the intention of doing so, this seems arbitrary, as this has not mentioned in the objectives.

**Thank you for the observation. This has been clarified in the objectives hence it was not arbitrary. The objective thus reads, to find out proximate determinants of adolescent fertility outcome (aggregate analysis and disaggregation by place of residence).**

Discussion

• So, do you mean that even after 20 years of Bledsoe and Cohen, 1993 analysis, still the fertility figures are same? Justify this. Expunch and put more recent literature

**Thanks for the observation. This has been expunched and more recent literature included.**

• Discussion and conclusion sections are clear, it would be better to come up with separate section on strengths and limitations.

**Thank you for the proposal. We have added a sub-section on Strengths and Limitations Just before the Conclusion.**

Why the author mentioned as “we”? Do you have any other author contributions?

**Yes. The article has three authors they include Naomi Monari, Alfred Agwanda and James Orwa. A section on author contributions has been inserted.**

**Author Contributions**

**Conceptualization:** Naomi Monari, Alfred Agwanda

**Data Analysis and interpretation:** Naomi Monari, James Orwa,

**Methodology:** Naomi Monari, Alfred Agwanda,

**Supervision:** Alfred Agwanda

**Validation:** Alfred Agwanda, James Orwa

**Writing original draft:** Naomi Monari

**Writing, review and editing:** Naomi Monari, Alfred Agwanda and James Orwa

6. PLOS authors have the option to publish the peer review history of their article ([what does this mean?](https://journals.plos.org/plosone/s/editorial-and-peer-review-process#loc-peer-review-history)). If published, this will include your full peer review and any attached files.


If you choose “no”, your identity will remain anonymous but your review may still be made public.


**Do you want your identity to be public for this peer review?** For information about this choice, including consent withdrawal, please see our [Privacy Policy](https://www.plos.org/privacy-policy).

Reviewer #1: No

Reviewer #2: **Yes:** Gayathri Abeywickrama

[NOTE: If reviewer comments were submitted as an attachment file, they will be attached to this email and accessible via the submission site. Please log into your account, locate the manuscript record, and check for the action link "View Attachments". If this link does not appear, there are no attachment files.]

While revising your submission, please upload your figure files to the Preflight Analysis and Conversion Engine (PACE) digital diagnostic tool, <https://pacev2.apexcovantage.com/>. PACE helps ensure that figures meet PLOS requirements. To use PACE, you must first register as a user. Registration is free. Then, login and navigate to the UPLOAD tab, where you will find detailed instructions on how to use the tool. If you encounter any issues or have any questions when using PACE, please email PLOS at [figures@plos.org](mailto:figures@plos.org). Please note that Supporting Information files do not need this step.
